# Transcatheter aortic valve replacement via a transsubclavian approach in a patient with severe aortic stenosis who had previously undergone kidney transplantation: A case report

**DOI:** 10.1097/MD.0000000000027210

**Published:** 2021-10-01

**Authors:** Seok Oh, Ju Han Kim, Dae Young Hyun, Kyung Hoon Cho, Min Chul Kim, Doo Sun Sim, Young Joon Hong, Youngkeun Ahn, Myung Ho Jeong, Kyo Seon Lee

**Affiliations:** a Department of Cardiology, Chonnam National University Hospital, Gwangju, Korea; b Department of Thoracic and Cardiovascular Surgery, Chonnam National University Hospital, Gwangju, Korea.

**Keywords:** aortic valve stenosis, kidney transplantation, transcatheter aortic valve replacement

## Abstract

**Rationale:**

Although the transfemoral approach is the gold standard for transcatheter aortic valve replacement (TAVR), it is not feasible in a considerable number of patients. We report a case of successful transsubclavian TAVR (TS-TAVR) in a patient with severe aortic stenosis (AS) who was ineligible for transfemoral TAVR because she was a kidney transplant recipient.

**Patient concerns:**

A 72-year-old Korean woman, who had previously undergone kidney transplantation in the right iliac fossa for end-stage kidney disease, was admitted to our center with dyspnea. Upon auscultation, grade IV systolic murmurs were detected in both upper sternal borders and the left lower sternal border, suggestive of valvular heart disease.

**Diagnosis:**

Two-dimensional transthoracic echocardiography revealed heavy calcification of the aortic valve with a high peak velocity (4.54 m/s) and mean pressure gradient (48.49 mm Hg), indicative of severe AS.

**Interventions:**

TS-TAVR was performed by a heart team comprised of interventional cardiologists, cardiac surgeons, and anesthesiologists. A self-expandable valve prosthesis (CoreValve^TM^ Evolut R^TM^, Medtronic Inc., Minneapolis, MN) was successfully deployed via the left subclavian artery.

**Outcomes:**

Post-TAVR 2-dimensional transthoracic echocardiography demonstrated a well-functioning valve with mild paravalvular leakage. The peak velocity had declined from 4.54 m/s to 2.22 to 2.24 m/s, and the mean pressure gradient had declined from 48.49 to 8.57–9.61 mmHg. The patient was discharged successfully and uneventfully.

**Lessons:**

Because kidney transplant recipients with severe AS are considered poor candidates for transfemoral TAVR, TS-TAVR is a suitable alternative to consider.

## 1 Introduction

Aortic stenosis (AS) is one of the most common valvular heart diseases in the older population, with a prevalence of approximately 5.2% in people aged ≥75 years.^[1]^ This degenerative disease is characterized by thickening and calcification of the aortic valve (AoV), which limits the motion of the valvular leaflets.^[1,2]^ End-stage AS causes obstruction of the left ventricular outflow tract, resulting in a decrease in cardiac output and exercise capacity, overt heart failure, and cardiac death. Symptomatic AS is associated with high mortality in the absence of prompt AoV replacement.^[3]^

Since the first-in-human successful transcatheter aortic valve replacement (TAVR) was performed in 2002,^[4]^ there has been a dramatic shift in the landscape of the treatment of severe AS.^[5]^ TAVR is a suitable alternative to surgical aortic valve replacement (SAVR) in patients with severe symptomatic AS. Certain landmark randomized clinical trials have demonstrated that TAVR is either superior or non-inferior to SAVR in patients with high^[6,7]^ or intermediate surgical risk,^[8,9]^ which resulted in the expansion of TAVR indications to a larger population of patients with severe AS. Certain pivotal trials have demonstrated that TAVR may also be indicated in patients with low surgical risk,^[10,11]^ which was consequently reflected in the 2020 guidelines.^[12]^

Because the percutaneous transfemoral approach is the least invasive route in TAVR, transfemoral TAVR (TF-TAVR) is considered the approach of choice for most patients.^[13]^ However, there are a considerable number of patients who are ineligible for TF-TAVR because of poor vascular status, high tortuosity of the aorta, or prior surgical interventions. In these clinical circumstances, transsubclavian TAVR (TS-TAVR) is a viable alternative.^[14]^

Here, we describe a case of a patient with severe AS who was not a candidate for the transfemoral approach because of prior kidney transplantation and was successfully treated with TS-TAVR.

## 2 Case report

**Figure 1:**
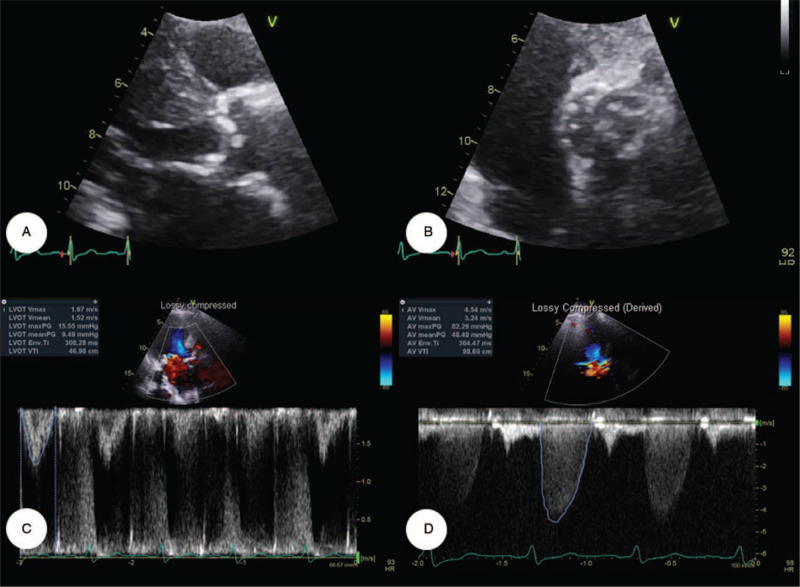
Initial transthoracic echocardiography. (A, B) Parasternal long- and short-axis views revealed a heavily calcified aortic valve. (C, D) The peak velocity was 4.54 m/s and the mean pressure gradient was 48.5 mm Hg, suggestive of severe aortic stenosis.

A 72-year-old Korean woman visited our tertiary center with the chief complaint of dyspnea (New York Heart Association functional class III). She had hypertension, diabetes mellitus, unstable angina, and chronic kidney disease. The patient had received percutaneous coronary intervention and had undergone kidney transplantation for end-stage kidney disease. Her dyspnea gradually worsened, which led her to visit our hospital for diagnosis and management. Her temperature was 36.5°C, her heart rate was 90 beats/min, her respiratory rate was 20 breaths/min, and her blood pressure was 132/50 mm Hg. Upon auscultation, grade IV systolic murmurs were detected in both upper sternal borders and the left lower sternal border. The high-sensitivity troponin-I concentration was 0.080 ng/mL (reference range, 0–0.05 ng/mL), the high-sensitivity troponin-T concentration was 0.386 ng/mL (reference range, 0–0.014 ng/mL), and the N-terminal pro-brain natriuretic peptide concentration was 29,234 pg/mL (reference range, 0–125 pg/mL). Two-dimensional transthoracic echocardiography demonstrated heavy calcification of the AoV, a peak velocity of 4.54 m/s, and a mean pressure gradient of 48.49 mm Hg (Fig. 1). Given these clinical manifestations, the patient was diagnosed with AS and concomitant acute decompensated heart failure. We decided to perform the optimal medical therapy for heart failure.

After medical treatment, including oxygen therapy and intravenous furosemide injection, the patient's clinical symptoms were relatively stable. A multidisciplinary heart team, comprised of interventional cardiologists, cardiac surgeons, cardiovascular imaging specialists, and anesthesiologists, reviewed the patient's clinical information. Her risk of mortality according to the European System for Cardiac Operative Risk Evaluation II was 27.23% (high surgical risk), and her Society of Thoracic Surgeons Predictive Risk of Mortality was 5.809% (intermediate surgical risk). In the heart-team conference, TAVR was determined as the optimal treatment for this patient.

**Figure 2:**
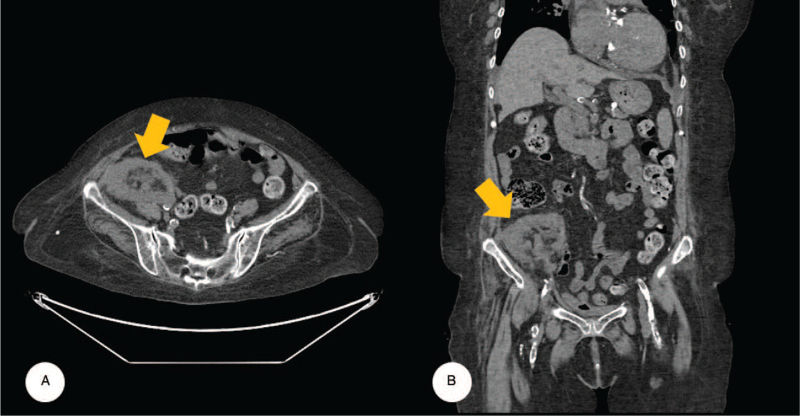
(A) Transverse, and (B) coronal views of the abdomen-pelvis computed tomography demonstrated mild atrophic change in the transplanted kidney at the right iliac fossa (arrows).

While the patient was in a clinically stabilized state, evaluation of her coronary and peripheral vascular status was commenced. Coronary computed tomography (CT) angiography demonstrated 2 well-deployed drug-eluting stents, one in the left anterior descending coronary artery and one in the left circumflex coronary artery, with visible distal antegrade runoff (see Figure S1, Supplemental Digital Content, Videos S1–S4, Supplemental Digital Content). Abdomen-pelvis CT demonstrated an atrophic change in the transplanted kidney in the right iliac fossa, and chronically atrophied native kidneys (Fig. 2, see Video S5, Supplemental Digital Content). As the transplanted kidney was at high risk for ischemic damage caused by TF-TAVR, we planned to use a transsubclavian approach instead.

**Figure 3:**
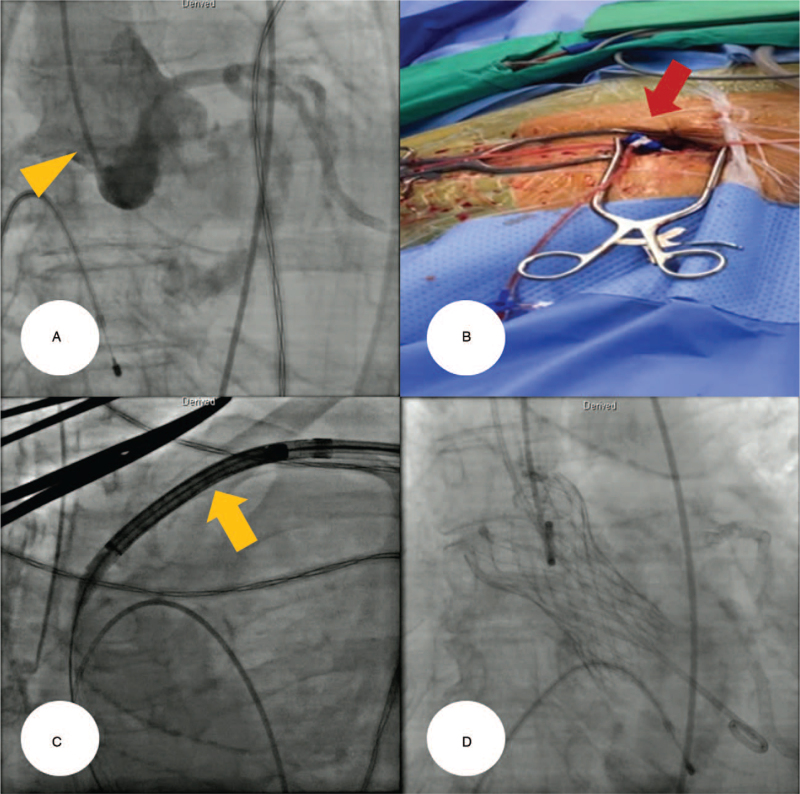
(A) A 5-Fr pigtail catheter (yellowish arrowhead) was positioned in the ascending aorta, in preparation to perform an aortogram. A temporary pacemaker wire was placed in the right ventricle via the femoral vein to enable backup artificial pacing in case of high-degree or complete atrioventricular block during the TAVR procedure. (B) The proximal left axillary artery was exposed with a surgical incision, and a 7-Fr sheath (red arrow) was inserted into the subclavian artery. (C, D) A 29-mm self-expandable valve prosthesis (CoreValve^TM^ Evolut R^TM^, Medtronic Inc., Minneapolis, MN) (yellowish arrow) was introduced via the left subclavian artery and successfully deployed at the annulus of the aortic valve. The final aortogram demonstrated satisfactory expansion of the valve prosthesis. TAVR = transcatheter aortic valve replacement.

The procedure was performed in a cardiac catheterization laboratory under local anesthesia plus conscious sedation. A 5-Fr pigtail catheter was introduced via the right femoral artery and placed in the ascending aorta. The initial aortogram demonstrated that the ascending aorta was of a sufficient length to perform TAVR. A temporary pacemaker wire was placed in the right ventricle via the femoral vein to enable backup artificial pacing in case of high-degree or complete atrioventricular block during the TAVR procedure (Fig. 3A). Thereafter, the proximal left axillary artery was exposed with a surgical incision in the deltopectoral groove, and a 7-Fr sheath was inserted into the subclavian artery (Fig. 3B). A 0.035-inch Amplatz Super Stiff^TM^ guidewire (Boston Scientific Inc., Marlborough, MA) was advanced into the left ventricle using the catheter-exchange technique (see Video S6, Supplemental Digital Content). Under fluoroscopic guidance, a 29-mm self-expandable valve prosthesis (CoreValve^TM^ Evolut R^TM^, Medtronic Inc., Minneapolis, MN) was introduced via the vascular access site of the left subclavian artery and slowly deployed at the annulus of the AoV (Fig. 3C, see Videos S7, S8, S9, Supplemental Digital Content). An immediate post-TAVR aortogram demonstrated satisfactory expansion of the valve prosthesis with mild paravalvular leakage (Fig. 3D) (see Video S10, Supplemental Digital Content). The peak-to-peak pressure gradient declined from 85 to 5 mm Hg (Figure S2, Supplemental Digital Content). The vascular access site in the left subclavian artery was surgically closed without any complications. In the TAVR procedure, 240 mL iso-osmolar contrast media (iodixanol; Visipaque^TM^, GE Healthcare, Princeton, NJ) was used, and the total fluoroscopic time was 70 minutes.

**Figure 4:**
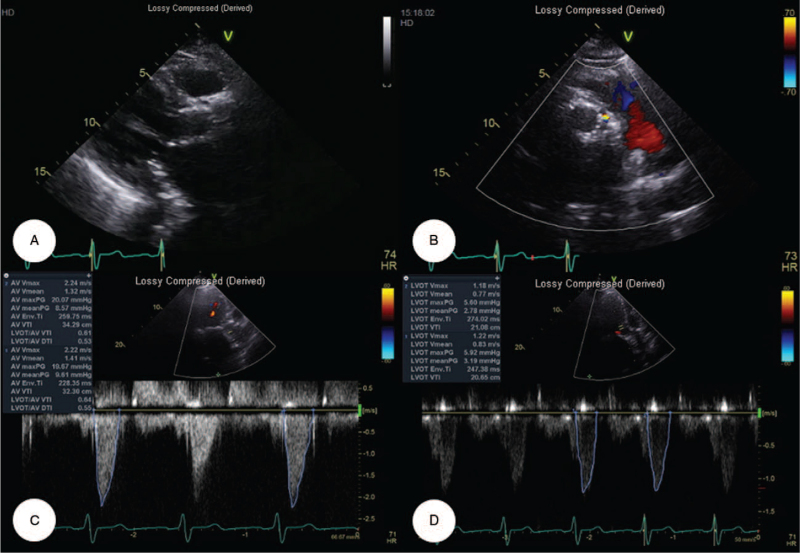
Follow-up transthoracic echocardiography. (A, B) Parasternal long- and short-axis views revealed that the valve prosthesis (CoreValve^TM^ Evolut R^TM^, Medtronic Inc., Minneapolis, MN) was satisfactorily expanded and apposed, with mild paravalvular leakage. (C, D) Peak velocity had declined from the initial 4.54 to 2.22–2.24 m/s, and the mean pressure gradient had also declined from 48.5 to 8.57–9.61 mm Hg.

After the procedure, the patient was transferred to the intensive care unit for hemodynamic monitoring and was maintained with the optimal medical therapy: antiplatelet therapy (aspirin, 100 mg/day and clopidogrel, 75 mg/day), a statin (atorvastatin, 20 mg/day), a beta-blocker (nebivolol, 2.5 mg/day), and an angiotensin II receptor blocker (valsartan, 80 mg/day). Several days later, the patient was transferred to the general ward. After 1 week, follow-up 2-dimensional transthoracic echocardiography demonstrated a well-functioning valve with mild paravalvular leakage (Fig. 4). The peak velocity had declined from 4.54 to 2.22–2.24 m/s, and the mean pressure gradient had also declined from 48.5 to 8.57–9.61 mm Hg. Two weeks after the TAVR procedure, the patient was successfully and uneventfully discharged from our hospital.

## 3 Discussion

In the modern era, TAVR is an effective treatment for patients with severe AS, regardless of their estimated surgical risk.^[15]^ In a meta-analysis of randomized clinical trials, the mortality rate for patients who underwent TF-TAVR was lower than for those who underwent SAVR, and TF-TAVR yielded lower risks of stroke, major bleeding, and atrial fibrillation, as well as a shorter hospital stay.^[16]^ Many approaches for TAVR have been introduced, including transfemoral,^[17]^ transapical,^[18]^ transaortic,^[19]^ and transsubclavian routes.^[20]^ Among them, the transfemoral approach is the current gold standard for TAVR and is used for first-line access.^[12]^ In contemporary guidelines, the feasibility of this vascular access route is one of the main features to be assessed when choosing between TAVR and SAVR.^[12]^ Hence, during pre-procedural testing, peripheral CT is needed. Nevertheless, TF-TAVR is not feasible in at least 10% to 15% of TAVR-eligible patients,^[21]^ because of anatomical contraindications such as aortoiliac tortuosity and calcification.^[21–23]^ Among the non-femoral access routes, the transsubclavian approach has certain technical advantages compared with the transapical and transaortic approaches. TS-TAVR is the least invasive non-femoral method because it involves no exposure of the pleural or pericardial spaces, no sternotomy, and no direct manipulation of the myocardium.^[15]^ Moreover, the other 2 procedures are more difficult and dangerous to perform because of their greater levels of invasiveness (as they involve thoracotomy) and associated complications.^[24,25]^ A comparative study based on data from the UK Transcatheter Aortic Valve Implantation registry demonstrated that both transapical and transaortic TAVR had worse outcomes than TF-TAVR, but that outcomes did not differ between TS-TAVR and TF-TAVR, leading the authors to conclude that TS-TAVR may be the safest non-femoral approach to TAVR.^[25]^ According to a large-scale, multicenter, observational study conducted in France from 2013 to 2017, both TS-TAVR and transcarotid TAVR yielded similar clinical outcomes to TF-TAVR, except in that the former approaches yielded a 2-fold lower rate of major vascular complications and unplanned vascular repairs.^[26]^

In this case report, successful TAVR in a kidney transplant recipient (KTR) with severe AS was described. Kidneys are one of the most commonly transplanted organs worldwide,^[27]^ and, as in this case, the right iliac fossa is often the preferred site for such transplantation because the iliac vessels are more superficial and larger in the right than in the left iliac fossa. Because the transplanted kidney was connected directly to the right common iliac artery with end-to-side arterial anastomosis, TF-TAVR might have induced a decrease in blood flow to this vascular access site, leading to structural and functional renal hypoperfusion and ischemic kidney damage. Hence, we performed TS-TAVR, with demonstrable success.

Generally, KTRs are considered at high risk for heart surgery. They commonly have poor outcomes after cardiac surgery,^[28]^ which may be mainly due to comorbid conditions, impaired renal function, and use of immunosuppressive agents.^[29]^ A retrospective, nationwide cohort study in the USA revealed that TAVR is a safe alternative to SAVR, with favorable short-term outcomes, in patients who had previously undergone kidney transplantation.^[30]^ Although the TAVR group was older and had a higher proportion of comorbidities than the SAVR group, TAVR was associated with lower rates of in-hospital complications and in-hospital mortality. The risks of acute kidney injury and infective endocarditis were statistically significantly lower in the TAVR group than in the SAVR group. In addition, the TAVR group had a shorter hospital stay, lower rates of discharge with disability, and lower 30-day re-hospitalization rates. Unfortunately, because the database that was used in that study lacked detailed procedural information, such as information about the vascular access sites, the authors could not compare the clinical outcomes of TAVR in KTRs depending on the access sites.

In the present case, TAVR was successfully performed via a multifaceted approach to prevent ischemic damage to the transplanted kidney. First, TAVR was selected as it is associated with a lower risk of acute kidney injury than SAVR is, as previously demonstrated.^[30]^ Second, as the subclavian route seemed less likely to cause ischemic damage to the transplanted kidney with lower rates of vascular complications than the conventional femoral route, TS-TAVR was considered a better choice than TF-TAVR in terms of the protection of renal function. Third, we chose a self-expandable rather than a balloon-expandable valve prosthesis. Because a self-expandable valve prosthesis does not require rapid ventricular pacing, which can create transient cardiac standstill, we predicted that it would result in a smaller degree of renal impairment than a balloon-expandable valve prosthesis. Fourth, we prevented the development of contrast-induced nephropathy by using iso-osmolar rather than low-osmolar contrast media.

To the best of our knowledge, this is the first reported case of TS-TAVR performed on a KTR with severe AS. Although the use of the subclavian artery as a vascular access site for TAVR is not novel, this report highlights that KTRs are poor candidates for TF-TAVR and that TS-TAVR is a useful alternative. There is a lack of clinical information about the safety and efficacy of TS-TAVR in this population; thus, large-scale, multicenter clinical trials are needed to fill this knowledge gap in future.

### 3.1 Conclusion

As patients who have undergone kidney transplantation in the right iliac fossa are considered poor candidates for the transfemoral approach in TAVR, the transsubclavian approach should be considered as an alternative with potential technical advantages to other non-femoral approaches.

## Author contributions

**Data curation:** Seok Oh, Ju Han Kim.

**Methodology:** Seok Oh, Dae Young Hyun, Kyo Seon Lee.

**Writing – original draft:** Seok Oh.

**Writing – review & editing:** Ju Han Kim, Dae Young Hyun, Kyung Hoon Cho, Min Chul Kim, Doo Sun Sim, Young Joon Hong, Youngkeun Ahn, Myung Ho Jeong.

## Supplementary Material

Supplemental Digital Content

## Supplementary Material

Supplemental Digital Content

## Supplementary Material

Supplemental Digital Content

## Supplementary Material

Supplemental Digital Content

## Supplementary Material

Supplemental Digital Content

## Supplementary Material

Supplemental Digital Content

## Supplementary Material

Supplemental Digital Content
