# Supplementary material for: Transcatheter aortic valve replacement via a transsubclavian approach in a patient with severe aortic stenosis who had previously undergone kidney transplantation: A case report
Source: Medicine (Baltimore). 2021 Oct 1;100(39):e27210. doi: 10.1097/MD.0000000000027210 (PMC8483856; doi:10.1097/MD.0000000000027210)

**Supplemental Fig. 1**. Coronary computed tomography angiography revealed two well-deployed drug-eluting stents in both the left anterior descending coronary artery and the left circumflex coronary artery, with visible distal runoff.


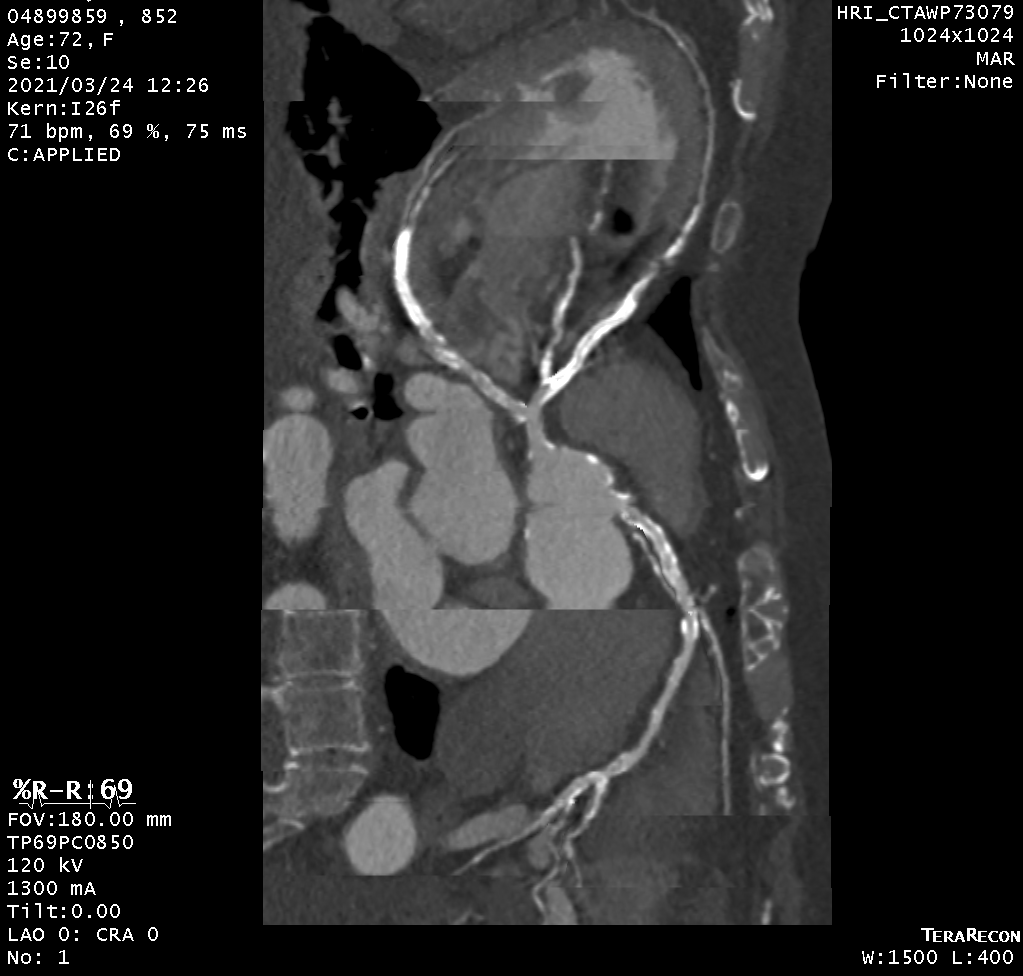

Supplement: Supplemental Digital Content [file medi-100-e27210-s001.doc]
