# Supplementary material for: Transcatheter aortic valve replacement via a transsubclavian approach in a patient with severe aortic stenosis who had previously undergone kidney transplantation: A case report
Source: Medicine (Baltimore). 2021 Oct 1;100(39):e27210. doi: 10.1097/MD.0000000000027210 (PMC8483856; doi:10.1097/MD.0000000000027210)
Supplement: Supplemental Digital Content [file medi-100-e27210-s002.doc]

**Supplemental Videos 1–4**. Coronary computed tomography angiography revealed two well-deployed drug-eluting stents in both the left anterior descending coronary artery and the left circumflex coronary artery, with visible distal runoff. Video 1, 9░s, 3.7 MB. Video 2, 9░s, 2.5 MB. Video 3, 12░s, 3.3 MB. Video 4, 12░s, 3.4 MB.
