# Supplementary material for: Transcatheter aortic valve replacement via a transsubclavian approach in a patient with severe aortic stenosis who had previously undergone kidney transplantation: A case report
Source: Medicine (Baltimore). 2021 Oct 1;100(39):e27210. doi: 10.1097/MD.0000000000027210 (PMC8483856; doi:10.1097/MD.0000000000027210)
Supplement: Supplemental Digital Content [file medi-100-e27210-s003.doc]

**Supplemental Video 5**. Abdomen-pelvis CT demonstrated atrophic change of the transplanted kidney at the right iliac fossa, and chronically atrophied native kidneys. 31░s, 2.8 MB.
