# Supplementary material for: Transcatheter aortic valve replacement via a transsubclavian approach in a patient with severe aortic stenosis who had previously undergone kidney transplantation: A case report
Source: Medicine (Baltimore). 2021 Oct 1;100(39):e27210. doi: 10.1097/MD.0000000000027210 (PMC8483856; doi:10.1097/MD.0000000000027210)
Supplement: Supplemental Digital Content [file medi-100-e27210-s004.doc]

**Supplemental Video 6**. The proximal left axillary artery was exposed with a surgical incision, and a 7-Fr sheath was inserted into the subclavian artery. A 0.035-inch Amplatz Super Stiff^TM^ guidewire (Boston Scientific Inc., Marlborough, MA, USA) was positioned in the left ventricle using a standard catheter-exchange technique. 2░min 51░s, 123 MB.
