# Supplementary material for: Transcatheter aortic valve replacement via a transsubclavian approach in a patient with severe aortic stenosis who had previously undergone kidney transplantation: A case report
Source: Medicine (Baltimore). 2021 Oct 1;100(39):e27210. doi: 10.1097/MD.0000000000027210 (PMC8483856; doi:10.1097/MD.0000000000027210)
Supplement: Supplemental Digital Content [file medi-100-e27210-s005.doc]

**Supplemental Video 7–9.** A 29-mm self-expandable valve prosthesis (CoreValve^TM^ Evolut R^TM^, Medtronic Inc., Minneapolis, MN, USA) was introduced via the vascular access site of the left subclavian artery and slowly deployed at the annulus of the aortic valve. Video 7, 30░s, 4.6 MB. Video 8, 23░s, 3.6 MB. Video 9, 8░min 8░s, 353 MB.
