# Supplementary material for: Transcatheter aortic valve replacement via a transsubclavian approach in a patient with severe aortic stenosis who had previously undergone kidney transplantation: A case report
Source: Medicine (Baltimore). 2021 Oct 1;100(39):e27210. doi: 10.1097/MD.0000000000027210 (PMC8483856; doi:10.1097/MD.0000000000027210)
Supplement: Supplemental Digital Content [file medi-100-e27210-s006.doc]

**Supplemental Video 10**. The final aortogram demonstrated satisfactory expansion of the valve prosthesis with trivial paravalvular leakage. 5░s, 1.0 MB.
