# Supplementary material for: Transcatheter aortic valve replacement via a transsubclavian approach in a patient with severe aortic stenosis who had previously undergone kidney transplantation: A case report
Source: Medicine (Baltimore). 2021 Oct 1;100(39):e27210. doi: 10.1097/MD.0000000000027210 (PMC8483856; doi:10.1097/MD.0000000000027210)

**Supplemental Fig. 2**. The peak-to-peak pressure gradient dramatically declined from 85░mmHg before TAVR to 5░mmHg after TAVR. TAVR, transcatheter aortic valve replacement


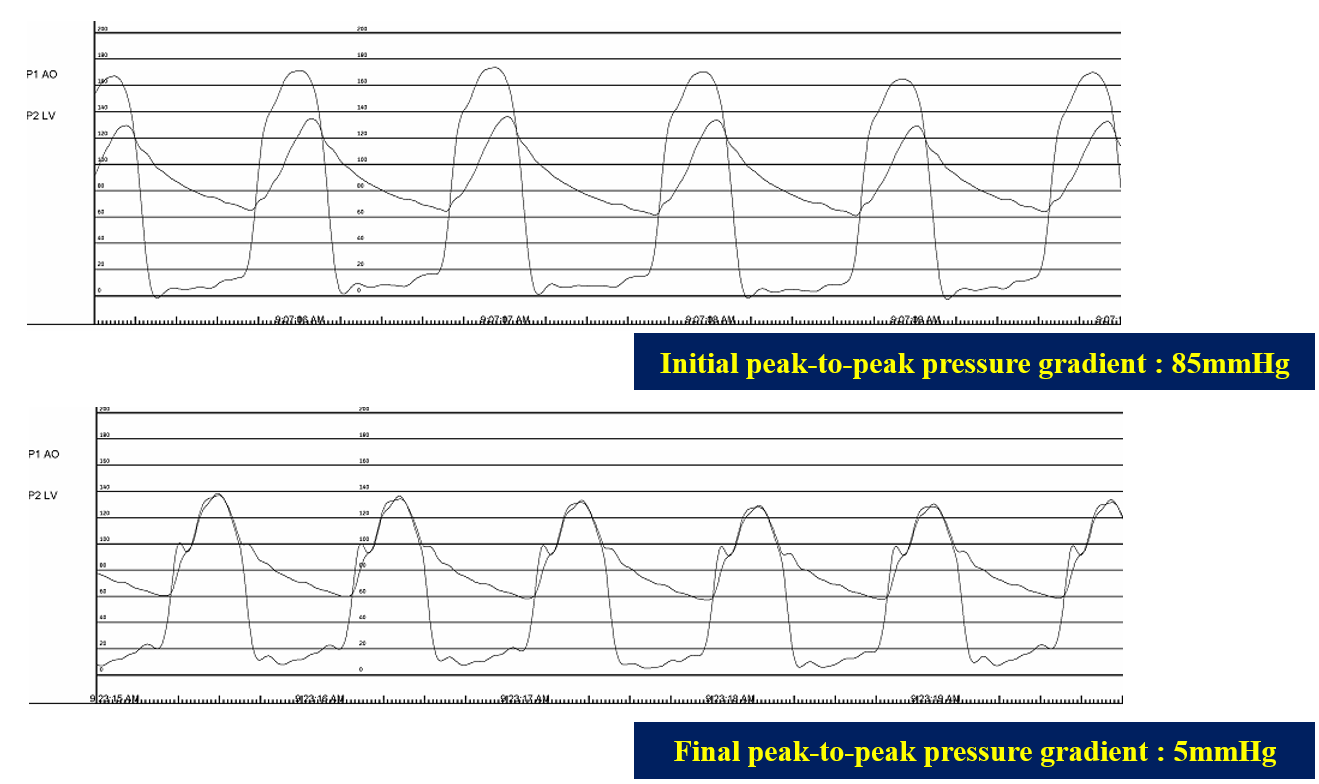

Supplement: Supplemental Digital Content [file medi-100-e27210-s007.doc]
